# Polycationic Peptide Engineering of Phage Endolysins Expands Host Range and Enhances Antibacterial and Antibiofilm Activities Against *Bacillus* Species

**DOI:** 10.1002/bit.70293

**Published:** 2026-07-06

**Authors:** Farhat Ansari, Tarushi, Vandan Nagar

**Affiliations:** ^1^ Food Technology Division, Bhabha Atomic Research Centre Mumbai India; ^2^ Homi Bhabha National Institute Mumbai India; ^3^ Protein Crystallography Section, Bhabha Atomic Research Centre Mumbai India

**Keywords:** artilysin, *Bacillus*, bacteriophage, Cecropin A, Endolysin, PCNP

## Abstract

The exponential rise in antimicrobial resistance has highlighted the urgent need for the development of alternative antibacterial strategies, including phage‐derived and engineered protein agents such as endolysins and artilysins. In this study, we report the rational design, expression, and functional characterization of novel artilysins derived from P19_358 lysin, a glycoside hydrolase family 24 enzyme. The native P19_358 lysin exhibits limited antibacterial activity, primarily against *Bacillus subtilis*, and requires EDTA pre‐treatment to act against Gram‐negative bacteria. To overcome this limitation, C‐terminal fusion constructs were engineered using polycationic peptides, including a Cecropin A‐derived peptide fragment and polycationic nonapeptide (PCNP), with and without flexible glycine‐serine linkers. Structural and biophysical analyses confirmed that peptide fusion did not interfere with the native catalytic domain. Functional assays demonstrated that the engineered artilysins exhibited enhanced antibacterial activity and an expanded host range compared to the native enzyme. Among these constructs, Cecropin A fused artilysins (Art1 and Art2) showed the highest bactericidal activity, achieving up to ~5.4 log_10_ reductions in *B. subtilis* MTCC 121 ^T^ and ~4.9 log_10_ reductions in *B. pumilus* MTCC 1640 ^T^ and *B. licheniformis* MTCC 429 ^T^. Additionally, Art2 exhibited pronounced antibiofilm activity, significantly reducing biofilm formation across the tested *Bacillus* species. Stability studies revealed that Art2 possessed superior thermal, pH, and salinity tolerance compared to the native lysin, retaining activity across a broad range of environmental conditions. These findings demonstrate that rational artilysin engineering can substantially enhance antibacterial potency, spectrum, and stability while preserving enzymatic function. Notably, Art2 emerges as a promising candidate for further development as a robust antimicrobial agent with potential applications in food safety and clinical settings.

## 1 Introduction

Antimicrobial resistance (AMR) represents one of the most serious global threats to modern medicine, causing 1.27 million direct and 5 million associated deaths globally in 2019. These numbers are projected to reach 10 million deaths annually by the year 2050 (Murray et al. 2022). The widespread use and overuse of antibiotics across human medicine, veterinary practice, livestock production, and aquaculture has accelerated the occurrence of multidrug‐resistant (MDR), extensively drug‐resistant (XDR), and pan‐drug‐resistant bacterial strains (Ventola 2015). These developments have severely compromised therapeutic options, while the high cost, complexity, and slow pace of novel antibiotic discovery further intensify the crisis (Gargate et al. 2025).

Phages offer high specificity and self‐amplifying activity; however, their clinical and practical applications face several challenges, including the potential development of phage resistance, limited host range, difficulties in dose standardization, and regulatory approvals (Abedon et al. 2011). In contrast, phage‐encoded endolysins have gained attention as robust antimicrobial agents due to their defined dosing, low cytotoxicity, rapid bactericidal action, and low probability of resistance development (Nelson et al. 2012). These peptidoglycan‐degrading enzymes are naturally produced during the phage lytic cycle to release progeny virions. When applied externally, endolysins exhibit potent antibacterial activity against Gram‐positive bacteria, owing to the direct accessibility of the peptidoglycan layer (Fischetti 2010). However, their efficacy against Gram‐negative bacteria is limited by the presence of an outer membrane, which acts as a permeability barrier. Recognizing their therapeutic potential, the World Health Organization (WHO) has classified endolysins as non‐traditional antimicrobials (Organization, W. H. 2024). Endolysin from phage DLn1 and *Pseudomonas* phage endolysin LysZC1 have demonstrated activity against *Bacillus cereus* and reduction of bacterial contamination in milk and lettuce, respectively (Abdelkader et al. 2022; Li et al. 2022). In addition, several pharmaceutical companies are actively advancing endolysin‐based therapeutics through clinical development. Notably, ContraFect (identifier: NCT04160468) and iNtRON Biopharma (identifier: NCT03089697) are in various stages of clinical trials of endolysin‐based agents (clinicalTrials.gov, accessed January 17, 2026).

Given that endolysins are naturally optimized to function from within bacterial cells, their external application against Gram‐negative bacteria requires strategies to overcome the outer membrane barrier. Recent advances in protein engineering have addressed this limitation through the development of artilysins, engineered endolysins fused with membrane‐disrupting peptides (Aitken et al. 2025) such as polycationic nonapeptides (PCNPs) enriched in arginine and lysine residues. This modification can improve outer membrane permeabilization in Gram‐negative bacteria and improve cell wall binding and lytic activity in Gram‐positive bacteria under varying environmental conditions, including changes in pH and ionic strength (Briers et al. 2014). These modifications significantly expand the host range and lytic efficacy of endolysins. *Aeromonas dhakensis* phage P19 encodes a glycoside hydrolase endolysin P19_358, which demonstrated lytic activity against Gram‐positive *B. subtilis* MTCC 121 ^T^ without EDTA pre‐treatment, and broad‐spectrum activity against multiple Gram‐negative pathogens following pre‐treatment with 100 mM EDTA. However, its narrow host range among Gram‐positive bacteria limited its broader application (Ansari and Nagar 2026).

In the present study, P19_358 lysin was engineered by conjugating polycationic peptides at its C‐terminus to generate a series of modified artilysins. The engineered artilysins demonstrated enhanced lytic activity, superior antibiofilm efficacy, and an expanded host range against *Bacillus* species. These findings underscore the potential of polycationic peptide engineering to transform phage endolysins into versatile and effective biocontrol agents with promising applications in food safety and infection management.

## 2 Material and Methods

### 2.1 Bacterial Strains and Culture Conditions

*Bacillus subtilis* MTCC 121 ^T^, *Bacillus pumilus* MTCC 1640 ^T^, and *Bacillus licheniformis* MTCC 429 ^T^ strains were procured from the Microbial Type Culture Collection (MTCC), India, and used for screening the antibacterial activity of the engineered artilysins. *Aeromonas dhakensis* CECT 5744 ^T^ was used as the host for propagation of phage P19 (Ansari and Nagar 2026). A complete list of bacterial strains used in the study is provided in Table S1. *Escherichia coli* DH5α and *E. coli* BL21 (DE3) were used for cloning and protein expression, respectively. All bacterial strains were routinely maintained in Luria Bertani (LB) medium, supplemented with kanamycin (50 µg/mL) where appropriate. Cultures were incubated at 37°C unless otherwise specified. For recombinant protein expression, cultures were incubated at 18°C following induction.

### 2.2 Concept, Design, 3D‐structure Prediction, and Comparison of Artilysins with P19_358 Lysin

P19_358 lysin is a 166 amino acid protein encoded by ORF**_**358 of phage P19 (Accession no. OM339718.1; protein id UKM62846.1). It has a molecular weight of 18.81 kDa, and is identified as a member of the baseplate hub subunit and tail lysozyme family (SUPERFAMILY: PHA02596), within glycoside hydrolase family 24 (IPR002196) (Ansari and Nagar 2026). The artilysin constructs were designed by genetically fusing cationic peptides: Cecropin A (KWKLFKKI) and PCNP (KRKKRKKRK) to the C‐terminal region of the P19_358 lysin in combination with and without three different glycine‐rich linkers (GSA, GSGS, and GS) (Table 1). Primers were designed to facilitate the incorporation of cationic peptides and linker sequences while maintaining the correct reading frame (Supplementary Table S2).

**Table 1:** Details of polycationic peptide and linker modifications of artilysins.

Artilysin	Linker	Peptide
P19_358 lysin	—	—
Art1	GSA	Cecropin A
Art2	GSGS	Cecropin A
Art3	—	Cecropin A
Art4	—	PCNP
Art5	GSGS	PCNP
Art6	GS	PCNP

The 3D structures of all artilysins were predicted using their amino acid sequences as input to the AlphaFold V3 server (Abramson et al. 2024) and visualised using PyMOL v2.0 (Schrödinger 2015). Structural alignment between P19_358 lysin and the engineered artilysins was performed using TM‐align, a widely used algorithm for protein structure comparison (Zhang 2005), by superimposing the structures of each artilysin onto that of P19_358 lysin. Root mean square deviation (RMSD), TM‐score, and MaxSub score were calculated to quantitatively evaluate structural conservation between P19_358 lysin and each engineered artilysin.

### 2.3 Cloning, Expression, and Purification of Artilysins

Genomic DNA of bacteriophage P19 was isolated following previously described protocols (Ansari and Nagar 2026). The endolysin gene was PCR amplified with Phusion High‐Fidelity DNA polymerase (New England Biolabs, USA) with gene‐specific primers containing *Bam*HI (GGATCC) and *Xho*I (CTCGAG) restriction sites at the N‐terminus and C‐terminus, respectively (Table S2). PCR products were purified and subjected to double digestion with the respective enzymes. In parallel, the pET‐28a (+) expression vector was digested with the same restriction enzymes. The digested inserts and vector were ligated using T4 DNA ligase and transformed into chemically competent *E. coli* DH5α cells via the heat‐shock method (Froger and Hall 2007). Positive transformants were screened by colony PCR followed by restriction digestion, and confirmed by Sanger sequencing. Confirmed recombinant plasmids were transformed into *E. coli* BL21(DE3) competent cells for protein expression. Transformants were cultured in LB medium containing kanamycin (50 µg mL^−1^) at 37°C until the optical density at 600 nm (OD_600_) reached 0.4–0.6. Protein expression was induced by the addition of isopropyl β‐D‐1‐thiogalactopyranoside (IPTG) to a final concentration of 0.5 mM.

Following induction, cultures were incubated at 18°C with shaking at 150–160 rpm for 16 h. Cells were harvested by centrifugation and resuspended in lysis buffer before being subjected to sonication for cell disruption. His‐tagged recombinant proteins were purified by Ni‐NTA affinity chromatography. Proteins were eluted using imidazole (up to 350 mM), and purity was qualitatively assessed by 15% SDS‐PAGE and quantitatively assessed using a NanoPhotometer NP80 (Implen, Germany). Purified artilysin proteins were dialysed against 1× phosphate‐buffered saline (PBS, pH 7.4) prior to biological and biochemical characterization.

### 2.4 Host Range of Phage P19, P19_358 Lysin and Artilysins

The host range of bacteriophage P19, P19_358 lysin, and engineered artilysins was evaluated using a spot assay as described by Kutter (2009). Briefly, overnight‐grown cultures of the test bacterial strains (Supplementary Table S1) were grown to the exponential phase and uniformly spread onto LB agar plates to form bacterial lawns. To assess the lytic activity of purified enzymes, 5 µL of purified P19_358 lysin or artilysin (5 µM) was dropped on a bacterial lawn for the spot test. Plates were incubated at 37°C for 16 h, after which zones of clearance were examined. The presence of a clear zone was recorded as a positive lytic reaction, whereas the absence of clearance was recorded as a negative result.

### 2.5 Optimal Dose Determination

The optimal dose of P19_358 lysin against *B. subtilis* MTCC 121 ^T^ was determined using a turbidity reduction assay as described by Nelson et al. (2012). Briefly, an overnight‐grown culture of *B. subtilis* MTCC 121 ^T^ was adjusted to an optical density at 600 nm (OD_600_) of 0.7. A total volume of 200 µL of the bacterial suspension, with different concentrations of endolysin (5.32‐0.16 µM), was dispensed into a 96‐well flat‐bottom microtiter plate (Himedia, India). Turbidity was monitored by measuring OD_600_ at 2‐min intervals for a total duration of 2 h using a microplate reader (BioTek Synergy H1 plate reader, Germany). Wells containing buffer instead of protein served as negative controls.

### 2.6 Antibacterial Activity Assessment By Turbidity Reduction Assay and Colony‐Forming Unit (CFU) Enumeration

The antibacterial activity of the P19_358 lysin and all engineered artilysins was evaluated against *B. subtilis* MTCC 121 ^T^, *B. pumilus* MTCC 1640 ^T^, and *B. licheniformis* MTCC 429 ^T^ using turbidity reduction and CFU enumeration assays as described by Son et al. (2021). For the turbidity reduction assay, overnight cultures of the test bacterial strains were resuspended in 1x PBS and adjusted to an OD_600_ of 0.7–0.75. Bacterial suspensions were treated with P19_358 lysin or individual artilysin at a final concentration of 5.32 µM in a total volume of 200 µL. Changes in turbidity were monitored by measuring OD_600_ at 20 min and 120 min using a microplate reader (BioTek Synergy H1 plate reader, Germany). Untreated samples receiving buffer alone served as negative controls. The relative antibacterial activity of P19_358 lysin and Art2 against EDTA‐pre‐treated *A. dhakensis* CECT 5744 ^T^ and *Salmonella* Typhimurium was also determined using a turbidity reduction assay (Chu et al. 2024). Briefly, bacterial cultures grown to the logarithmic phase were harvested by centrifugation, concentrated, and washed with 1x PBS. The bacterial pellets were then incubated with 100 mM EDTA for 10 min to permeabilize the outer membrane. Following EDTA treatment, the cells were washed twice with 1x PBS and incubated with either P19_358 lysin or Art2. Samples containing 1x PBS instead of lysin served as controls. Changes in turbidity were monitored by measuring OD_600_ at 0 min and 120 min and percentage lytic activity was calculated by equation 1.

(1) $$(\%)\;\text{Lytic}\;\text{activity}=\frac{O{\text{.D.}}_{\text{600nm}}\left(\text{control}\right)-O{\text{.D.}}_{\text{600nm}}\left(\text{P19}\_\text{358}\;\text{lysin}\;\text{or}\;\text{artilysin}\;\text{treated}\right)}{O{\text{.D.}}_{\text{600nm}}\{\text{initial}\;\left(\text{0}\;\text{min}\_\text{control}\right)\}}\times 100$$

For the CFU enumeration assay, bacterial cells were adjusted to approximately 1 × 10^7^ CFU mL^−1^ in 1× PBS. Cell suspensions were treated with P19_358 lysin or artilysin at a final concentration of 5.32 µM and incubated at 37°C for 2 h. Following incubation, samples were serially diluted and plated onto Luria agar (LA) plates. Plates were incubated overnight at 37°C, and surviving colonies were enumerated to determine CFU reduction relative to untreated controls.

### 2.7 Evaluation of Antibiofilm Activity of P19_358 Lysin and Artilysins

The ability of P19_358 lysin and engineered artilysins to inhibit biofilm formation was evaluated using a crystal violet (CV) staining method as described by Yu and Kong (2025a). Briefly, overnight cultures of *B. subtilis* MTCC 121 ^T^, *B. pumilus* MTCC 1640 ^T^, and *B. licheniformis* MTCC 429 ^T^ of OD_600_ to 1.0 were diluted (1:100) in fresh LB medium and dispensed into 96‐well flat‐bottom microtiter plates (Himedia, India). P19_358 lysin and engineered artilysins were added at a final concentration of 5.32 µM at the time of inoculation. Wells with PBS, instead of protein, served as negative controls. Plates were incubated under static conditions at 37°C for 24 h to allow biofilm development.

To evaluate the effect of lysins on mature biofilms, *B. subtilis* MTCC 121 ^T^ was allowed to form biofilms for 24 h under static conditions. After incubation, bacterial cells were removed, and the wells were gently washed once with 1x PBS. Subsequently, 200 µL of P19_358 lysin or artilysins (5.32 µM) was added to separate wells and incubated for an additional 4 h at 25°C. Wells containing 1x PBS instead of lysin served as controls. After incubation, bacterial cells were removed, and wells were gently washed twice with PBS. Biofilms were fixed with 2% (v/v) glutaraldehyde for 15 min, followed by washing to remove residual fixative. Plates were air‐dried for 30 min, and biofilms were stained with 1% (w/v) crystal violet, prepared in methanol, for 20 min. Excess stain was removed by washing, and the bound crystal violet was solubilized using an ethanol‐acetone (80:20) mixture. Absorbance was measured at 570 nm to quantify biofilm biomass. Biofilm inhibition was expressed as a percentage relative to the untreated control. Percentage of residual biofilm was calculated by following equation 2.

(2) $$\text{Residual}\;\text{biofilm}\;\left(\%\right)\;=\frac{O{\text{.D.}}_{\text{570nm}}\left(\text{control}\right)-O{\text{.D.}}_{\text{570nm}}\left(\text{P19}\_\text{358}\;\text{lysin}\;\text{or}\;\text{artilysin}\right)}{O{\text{.D.}}_{\text{570nm}}\left(\text{control}\right)}\times 100$$

### 2.8 Assessment of Stability of P19_358 Lysin and Artilysins under Different Temperature, Ph, and Salinity Conditions

The protein stability of P19_358 lysin and the selected artilysin (Art2) at different temperatures, pH, and salinity conditions was determined against *B. subtilis* MTCC 121 ^T^ according to Yu and Kong (2025a). For temperature stablity analysis, P19_358 lysin and Art2 were incubated at 4°C, 25°C, 35°C, 45°C, 55°C, and 65°C for 30 min. For pH stability, proteins were incubated across a pH range of 2‐12 using a universal buffer system. For salinity stability, proteins were tested at varying NaCl concentrations ranging from 0.1 to 1 M. For all stability assays, *B. subtilis* MTCC 121 ^T^ cells were adjusted to an optical density at 600 nm (OD_600_) of 0.7–0.75. Proteins were added at a final concentration of 5.32 µM. *B. subtilis* MTCC 121 ^T^ cells containing the appropriate buffer, instead of protein, served as the negative control. Turbidity was measured spectrophotometrically at 600 nm at 0 min and after 2 h of incubation. For pH and salt stability assays, *B. subtilis* MTCC 121 ^T^ cells with or without proteins were incubated at 25°C. Relative enzymatic activities were calculated based on the reduction in turbidity after 2 h compared to the untreated control, using the equation described below.

(3) $$\text{Relative}\;\text{activity}\;\left(\%\right)=\frac{\left\{\text{Initial}\;{\text{O.D.}}_{\text{600nm}}(\text{0}\;\text{min})-\text{Final}\;{\text{O.D.}}_{\text{600nm}}(\text{2}\;\text{h})\right\}}{\text{Maximum}\;\text{activity}\;\text{condition}\;\{\text{Initial}\;{\text{O.D.}}_{\text{600nm}}(\text{0}\;\text{min})-\text{Final}\;{\text{O.D.}}_{\text{600nm}}(\text{2}\;\text{h})\}}\times 100$$

### 2.9 Evaluation of Thermal Stability of P19_358 Lysin and Artilysins Across a pH Range By Differential Scanning Fluorimetry (DSF)

Thermal stability of P19_358 lysin and all artilysins across a pH range of 4–10 was determined by DSF analysis using the protocol described earlier (Tarushi et al. 2025). Briefly, the purified proteins were incubated in citrate‐HEPES‐CHES (CHC) buffer of varying pH, combined with SYPRO Orange dye (final concentration 5×), and allowed to equilibrate at 25°C for 30 s. The samples were then subjected to a temperature increase from 25°C to 95°C at a rate of 0.5°C per minute using a qTOWER3 Real‐Time PCR system (Analytik Jena, Germany). Fluorescence signals generated during protein unfolding were recorded, and T_m_ values were derived from the first derivative of the fluorescence intensity curves.

### 2.10 Circular Dichroism (CD) Spectroscopy

CD spectroscopy was performed to determine the secondary structural characteristics of P19_358 lysin and Art2 (Chakravarty et al. 2022). Purified proteins were prepared in a low‐ionic salt buffer (10 mM Tris‐HCl, 20 mM NaCl, pH 7.4) and analysed using a quartz cuvette with a path length of 1 mm. Spectra were recorded over a wavelength range of 200–250 nm using a Jasco J‐815 CD spectropolarimeter. Each spectrum represented the average of three scans, and baseline correction was carried out using the respective buffer. The obtained spectra were used to estimate secondary structural content.

### 2.11 Fluorescence Spectroscopy

Intrinsic fluorescence spectroscopy was used to determine any changes in protein folding and tertiary structural features resulting from artilysin modification. Measurements were carried out at room temperature using a fluorescence spectrophotometer (CLARIOstar multimode reader, BMG Labtech, Germany). Protein samples were excited at 280 nm, and emission spectra were recorded up to 500 nm. Changes in fluorescence intensity and shifts in emission maxima (λ_max_) were analysed to monitor conformational changes (Tarushi et al. 2025).

### 2.12 Scanning Electron Microscopy (SEM)

To evaluate cell morphological changes, bacterial cells were analysed using SEM. Exponentially grown cultures of *B. subtilis* MTCC 121 ^T^ and *B. pumilus* MTCC 1640 ^T^ were incubated with P19_358 lysin and Art2 for 1 h. Control samples consisted of bacterial cells treated with buffer alone instead of lysin. The cells were initially fixed overnight with 2.5% glutaraldehyde, followed by post‐fixation with 1% osmium tetroxide. Subsequently, the samples were washed with PBS and subjected to a stepwise dehydration process using increasing concentrations of ethanol up to 100%. The prepared samples were then examined using a Hitachi SU3800 SEM at an accelerating voltage of 30 kV, with imaging performed at magnifications of up to 25,000×.

### 2.13 Data Analysis

All experiments were performed in both technical and biological triplicates, and data are presented as mean ± standard deviation. Statistical significance (*p* < 0.05) was determined using one‐way or two‐way analysis of variance (ANOVA), depending on the experimental design. Post hoc comparisons between groups were conducted using Tukey's test. All statistical analyses were carried out using OriginPro 8.5 software.

## 3 Results

### 3.1 Conceptual Design, 3D Structures, and Structural Superposition of Artilysins

The native endolysin reported in this study was taken from ORF_358 of *A. dhakensis* phage P19 (Ansari and Nagar 2026) and it served as the parental enzyme for engineering the artilysins described herein. To improve antibacterial efficacy and expand the activity spectrum, artilysins were engineered by genetically fusing different cationic peptides (Cecropin A and PCNP) to the C‐terminal region of the P19_358 lysin. These fusions were designed either with or without glycine‐rich flexible linkers (GSA, GSGS, or GS) to evaluate the effect of linker length and flexibility on protein activity (Table 1).

Predicted 3D structures of the P19_358 lysin and engineered artilysins were compared to evaluate the structural impact of C‐terminal polycationic peptide fusion. Structural alignment revealed that all 166 residues of the native endolysin aligned across the artilysin structures, corresponding to almost the entire length of the native P19_358 lysin (Supplementary Figure S1). The alignment yielded a core RMSD ranging from 0.21 to 0.66 Å, indicating a high degree of structural similarity among all the proteins. TM‐scores ranged from 0.82 to 0.94, and MaxSub scores ranged from 0.75 to 0.93 (Table S3), confirming that the native endolysin and artilysins share the same global fold, and that artilysin modification has not interfered with the active site or groove of the proteins.

### 3.2 Expression and Purification of P19_358 Lysin and Artilysins

All artilysin genes were successfully cloned into the pET‐28a(+) vector and transformed into *E. coli* DH5α, confirmed by PCR amplification, restriction digestion, and Sanger sequencing (Supplementary Figure S2). Positive clones were transformed into *E. coli* BL21(DE3), cultured, and induced with IPTG at low temperature. Recombinant proteins were predominantly present in the soluble fraction and were purified using Ni–NTA affinity chromatography. SDS‐PAGE analysis confirmed the expression of all proteins at their expected molecular weights, with P19_358 lysin having a lower molecular weight as compared to artilysins (Supplementary Figure S3). Protein concentrations were quantified spectrophotometrically, and purified proteins were subsequently dialysed against 1× PBS and used for functional and stability analyses.

### 3.3 Optimal Dose Determination

**Figure 1:**
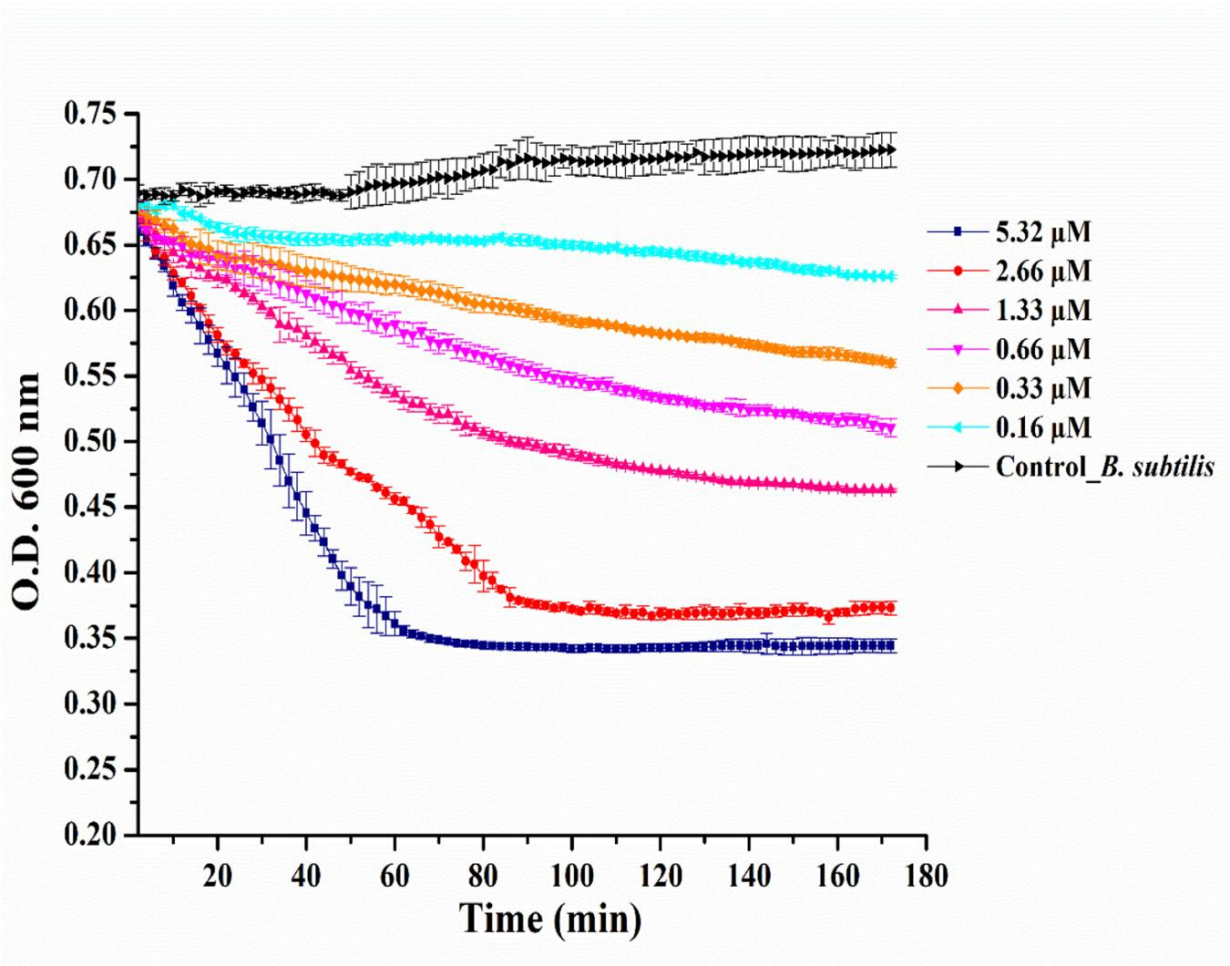
Optimal concentration determination of P19_358 lysin against *B. subtilis* MTCC 121 ^T^ by turbidity reduction assay. Absorbance was measured at 600 nm every 2 min for 3 h using different concentrations of P19_358 lysin; bacterial culture containing PBS was used as a control. Statistical significance was determined by two‐way ANOVA at *p* < 0.05.

Treatment of *B. subtilis* MTCC 121 ^T^ suspension with increasing concentrations of P19_358 lysin resulted in a concentration‐dependent decrease in OD_600_ over time. Higher concentrations of P19_358 lysin induced rapid and sustained turbidity reduction, whereas lower concentrations showed reduced or delayed lytic activity (Figure 1). Based on these observations, 5.32 µM was selected as the optimal working concentration for subsequent antibacterial and comparative studies, as it consistently produced maximal turbidity reduction within the experimental timeframe.

### 3.4 Host Spectrum Determination

The native P19_358 lysin demonstrated lytic activity only against *B. subtilis* MTCC 121 ^T^ among Gram‐positive bacteria and no activity against Gram‐negative bacteria in the absence of outer membrane permeabilization. (Supplementary Table S1). In contrast, all engineered artilysins efficiently lysed *B. subtilis* MTCC 121 ^T^, *B. pumilus* MTCC 1640 ^T^, and *B. licheniformis* MTCC 429 ^T^ without EDTA pre‐treatment among tested Gram‐positive and Gram‐negative bacteria (Supplementary Table S1, Supplementary Figure S4). These results demonstrate that fusion of polycationic peptides to the C‐terminus of P19_358 lysin expanded its host range and enabled effective lytic activity against multiple *Bacillus* species.

### 3.5 Antibacterial Activity Determination

Using the turbidity reduction assay, Art1 and Art2 achieved 64–66% relative antibacterial activity, and Art3 and Art6 achieved 55–58% within the initial 20 min, while all artilysins reached 75–86% relative activity by 2 h in *B. subtilis* MTCC 121 ^T^. In comparison, P19_358 showed only 16% relative activity at 20 min, and reached 69% by 2 h (Supplementary Figure S5A). In *B. pumilus* MTCC 1640 ^T^, Art2 achieved the highest relative activity of 61% at 20 min, followed by Art3 (51%) and Art1 and Art5 (24–28%), with all artilysins reaching 80–88% relative activity by 2 h. P19_358 lysin showed no significant activity against *B. pumilus* MTCC 1640 ^T^ at any time point (Supplementary Figure S5B). Similarly, in *B. licheniformis* MTCC 429 ^T^, the highest relative activity of 47% was achieved by Art2 in 20 min, and all artilysins achieved 71–80% by 2 h, whereas P19_358 lysin showed no significant activity as compared to the control (Supplementary Figure S5C).

**Figure 2:**
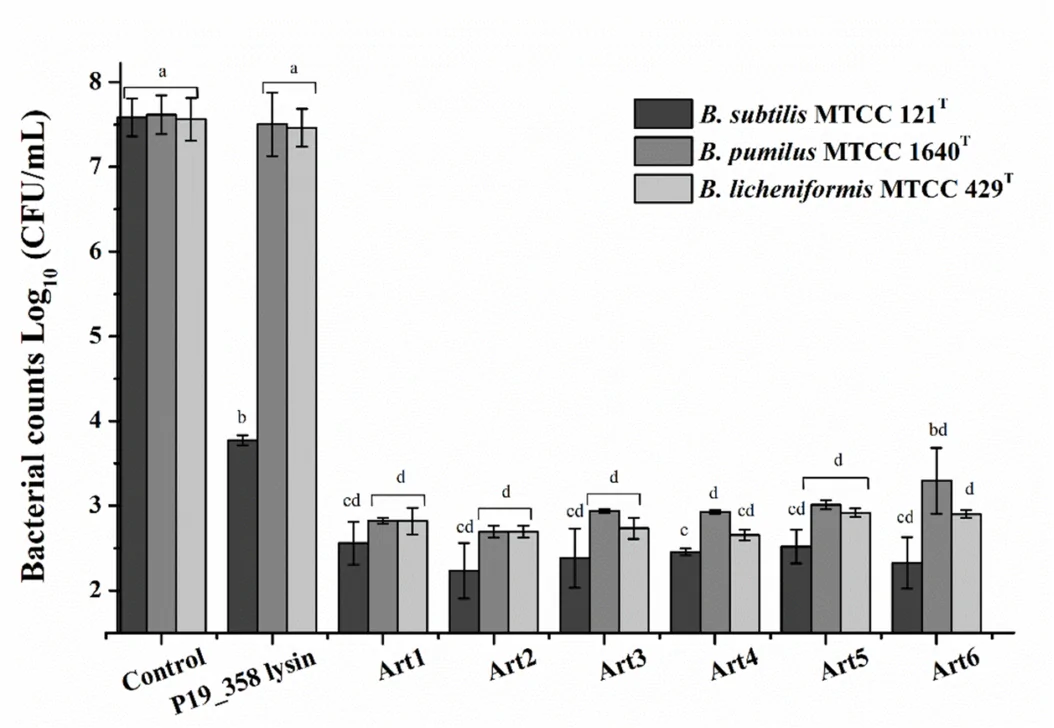
Lytic activity determination by colony‐forming unit (CFU) enumeration against *B. subtilis* MTCC 121 ^T^, *B. pumilus* MTCC 1640 ^T^, and *B. licheniformis* MTCC 429 ^T^ after 2 h. Statistical significance was determined by two‐way ANOVA at *p* < 0.05. Different letters above the error bars represent a significant difference in growth between strains treated with the same protein at *p* < 0.05, whereas the same letter represents no significant difference under the same condition.

No substantial differences in relative activity were observed between the P19_358 lysin and artilysin (Art2) when tested against EDTA‐pre‐treated *A. dhakensis* CECT 5744 ^T^ and *S*. Typhimurium. Following pre‐treatment with 100 mM EDTA, both P19_358 and the artilysins exhibited comparable antibacterial activity against the tested Gram‐negative bacteria (Supplementary Figure S6). CFU reduction assays further validated these findings. In *B. subtilis* MTCC 121 ^T^, P19_358 lysin resulted in approximately 3.9 log_10_ CFU reductions after 2 h, whereas Art2 caused a higher reduction of 5.4 log_10_ CFU, and the remaining artilysins produced reductions ranging from 4.8 to 5.3 log_10_ CFU within the same time (Figure 2). In *B. pumilus* MTCC 1640 ^T^, P19_358 lysin showed negligible bactericidal activity, whereas all artilysins caused significant reductions of approximately 4.4–4.9 log_10_ CFU. A similar trend was observed for *B. licheniformis* MTCC 429 ^T^, where P19_358 exhibited no detectable activity, while all artilysins resulted in 4.6–4.9 log_10_ CFU reductions after 2 h (Figure 2). Overall, all artilysins demonstrated consistent and robust antibacterial activity across all three *Bacillus* species. Although their performance was largely comparable, Art2 consistently exhibited slightly higher antibacterial efficacy and was therefore selected for subsequent stability and physicochemical characterization studies.

### 3.6 Anti‐Biofilm Activity Assessment

**Figure 3:**
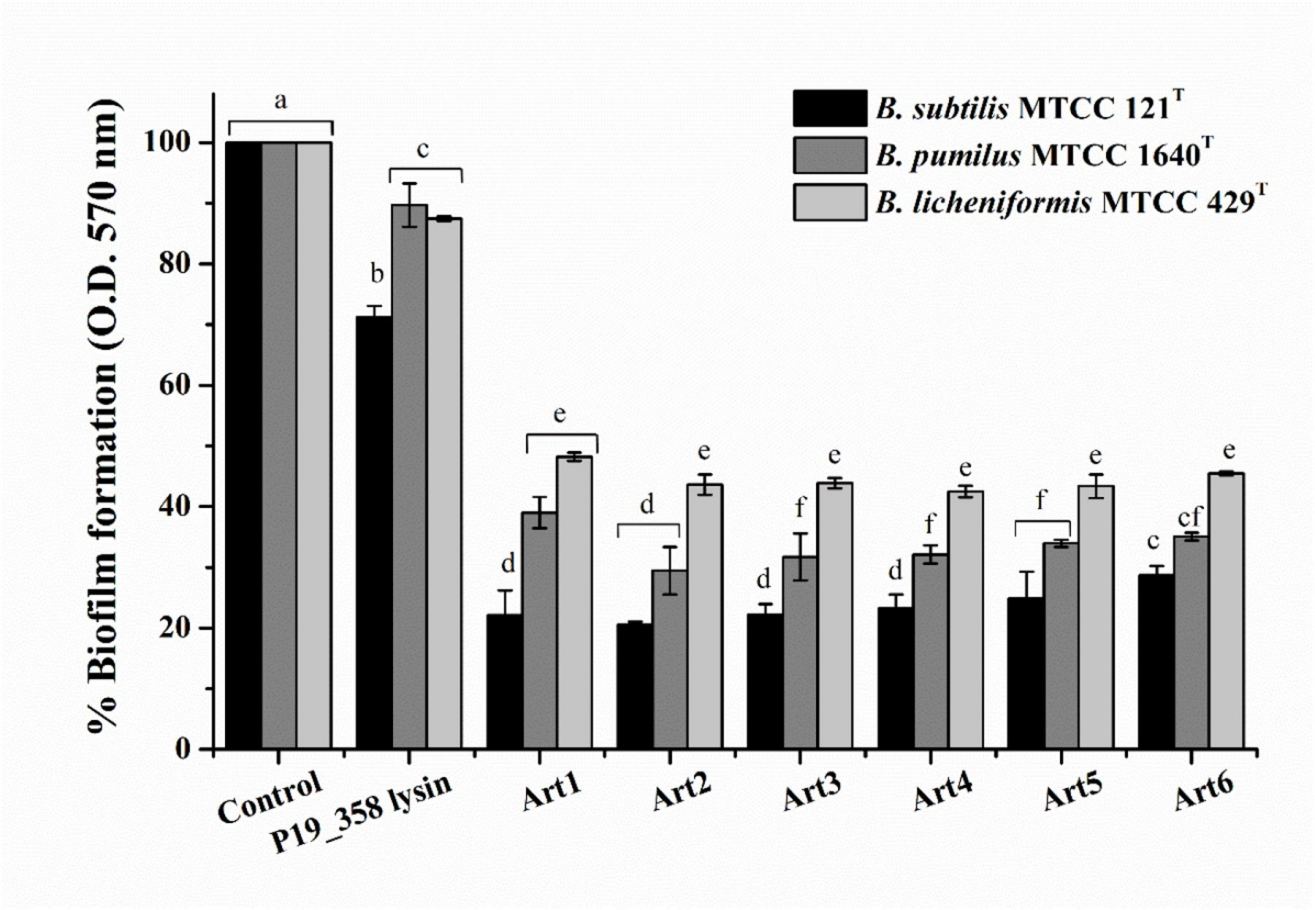
Percentage of biofilm formation by *B. subtilis* MTCC 121 ^T^, *B. pumilus* MTCC 1640 ^T^, and *B. licheniformis* MTCC 429 ^T^ treated with P19_358 lysin and artilysins determined by the crystal violet method. Statistical significance was determined by two‐way ANOVA at *p* < 0.05. Different letters above the error bars represent a significant difference in percentage biofilm formation between bacterial strains treated with P19_358 lysin and artilysins at *p* < 0.05, whereas the same letter represents no significant difference under the same condition.

Art2 treatment significantly reduced biofilm formation across all three *Bacillus* species (Figure 3). Relative biofilm formation by *B. subtilis* MTCC 121 ^T^ was reduced to approximately 20–28%, while *B. pumilus* MTCC 1640 ^T^ and *B. licheniformis* MTCC 429 ^T^ exhibited relative biofilm formation of approximately 30–40% and 43–48%, respectively, compared to untreated controls. In contrast, treatment with P19_358 lysin resulted in substantially higher residual biofilm formation (Figure 3). *B. subtilis* MTCC 121 ^T^ retained approximately 70% biofilm biomass, and no significant reduction in biofilm formation was observed for *B. pumilus* MTCC 1640 ^T^ and *B. licheniformis* MTCC 429 ^T^ relative to the control group (Figure 3). In contrast, when tested against pre‐formed mature biofilms of *B. subtilis* MTCC 121^T^, neither P19_358 lysin nor the engineered artilysins produced a comparable reduction in biofilm biomass relative to the untreated control (Supplementary Figure S7), indicating limited activity against established mature biofilms.

### 3.7 Stability Studies of P19_358 Lysin and Artilysins

**Figure 4:**
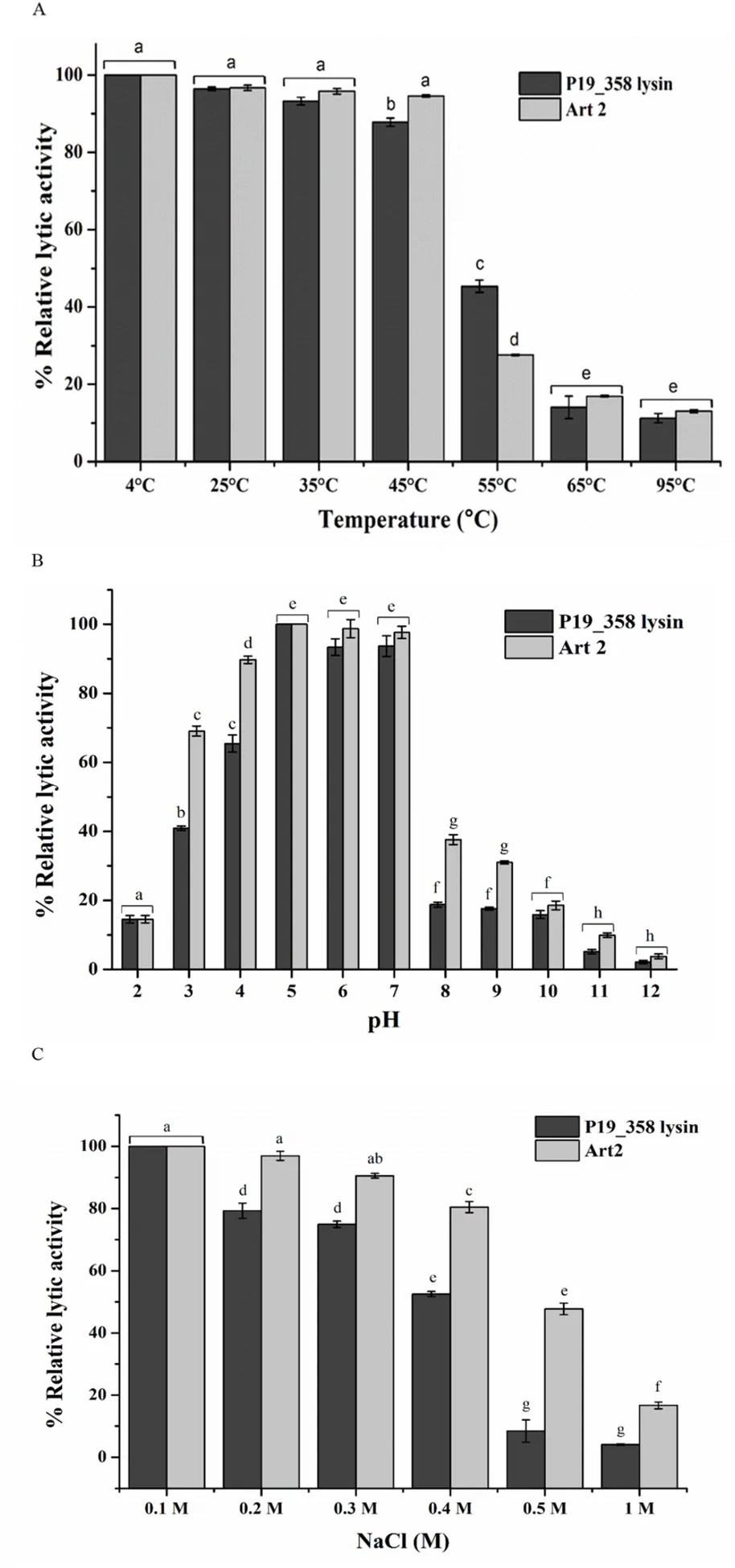
Stability assessment of P19_358 lysin and Art2 was assessed by determining the percentage relative lytic activity against *B. subtilis* MTCC 121 ^T^: (A) thermal stability (4°C‐95°C), (B) pH stability (pH 2–12), (C) salinity stability (NaCl concentration 0.1–1 M). Statistical significance was determined by two‐way ANOVA at *p* < 0.05. Different letters above the error bars represent a significant difference between the relative lytic activities of P19_358 lysin and Art2 under different stability conditions at *p* < 0.05, whereas the same letter represents no significant difference under the same condition.

Both P19_358 lysin and Art2 retained maximum lytic activity at 4°C and remained stable up to 45°C following 30 min of incubation at the respective temperatures. Up to 45°C, Art2 exhibited comparatively higher thermal stability than P19_358 endolysin, retaining greater relative activity at the elevated temperatures (Figure 4A). Beyond 45°C, a substantial decline in activity was observed for both the proteins (Figure 4A). Art2 retained 27% relative activity at 55°C, declining further to 13–16% at temperatures up to 95°C. Similarly, P19_358 lysin retained 45% relative activity at 55°C, declining to 11–14% at temperatures up to 95°C.

pH stability analysis demonstrated that both P19_358 and Art2 showed maximal activity at pH 5. Art2 maintained high relative activity (approximately 90–100%) across a broader pH range of pH 4–7, whereas P19_358 lysin displayed comparable stability over a narrower range of pH 5–7. At extreme acidic or alkaline pH values, a marked decrease in relative lytic activity was observed for both proteins (Figure 4B).

Salinity stability analysis revealed that both proteins exhibited the highest relative activity at 0.1 M NaCl. Beyond this concentration, Art2 maintained approximately 80% relative activity up to 0.4 M NaCl, whereas P19_358 lysin showed a notable decline, retaining only about 53% activity at the same concentration. At 0.5 M NaCl, Art2 still retained moderate activity of approximately 48%, while P19_358 lysin activity dropped sharply to just 8%. At 1 M NaCl, both proteins experienced a significant loss in relative lytic activity (Figure 4C).

### 3.8 pH‐Dependent Thermal Stability Profile

**Figure 5:**
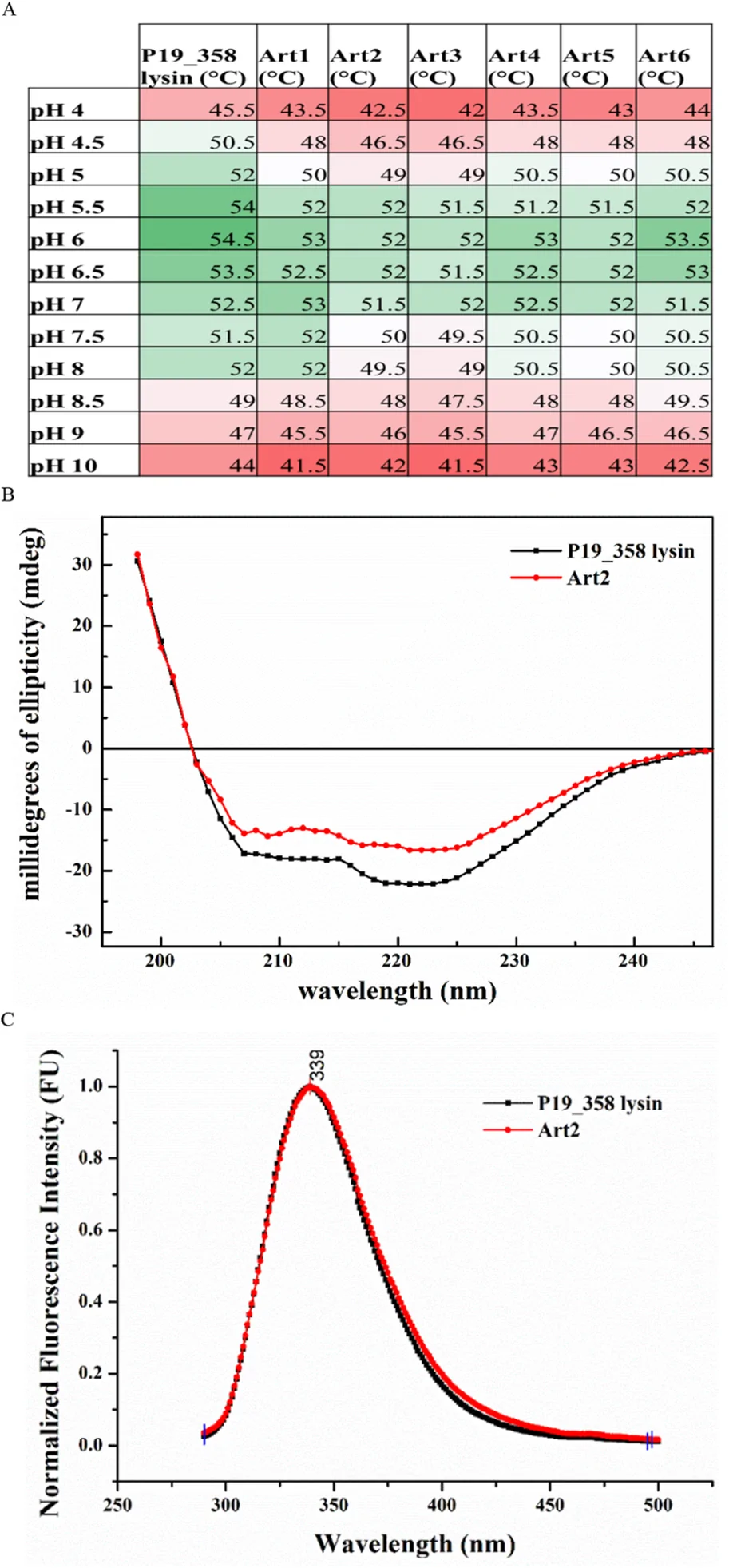
Physicochemical characterization of P19_358 lysin and artilysins. (A) thermal stability at different pH by DSF analysis of P19_358 lysin and artilysins, (B) CD spectroscopy of P19_358 lysin and Art2, and (C) fluorescence spectroscopy of P19_358 lysin and Art2.

The thermal stability of P19_358 lysin and all artilysins exhibited variations across the tested pH range (Figure 5A). Maximum stability was observed at pH 6, where all proteins showed the highest T_m_ values, indicating the most stable folded conformation. T_m_ values remained within 1°C of the maximum across a pH range of 5.5–7, suggesting moderate stability within this range. Overall, P19_358 lysin showed slightly higher T_m_ values as compared to artilysin under the same pH condition (Figure 5A).

### 3.9 Secondary Structural Profiling

CD spectroscopic analysis of P19_358 lysin and Art2 revealed well‐folded conformational structures predominantly characterized by α‐helical secondary structure (Figure 5B). Both proteins showed similar spectral patterns, suggesting that addition of C‐terminal extensions did not alter the overall conformation of the protein (Figure 5B).

### 3.10 Tertiary Structural Characterization

Intrinsic fluorescence spectra of P19_358 lysin and Art2 exhibited similar fluorescence emission profiles, with a maximum emission wavelength (λ_max_) at 339 nm for both proteins. This indicates that aromatic residues, particularly tryptophan, are located in a relatively similar microenvironment in both proteins, suggesting similar tertiary structures. The absence of any significant shift in λ_max_ or major changes in fluorescence intensity further indicates that C‐terminal polycationic peptide fusion did not substantially alter the internal conformation of the native P19_358 lysin (Figure 5C).

### 3.11 SEM Micrographs Illustrating Morphological Changes

**Figure 6:**
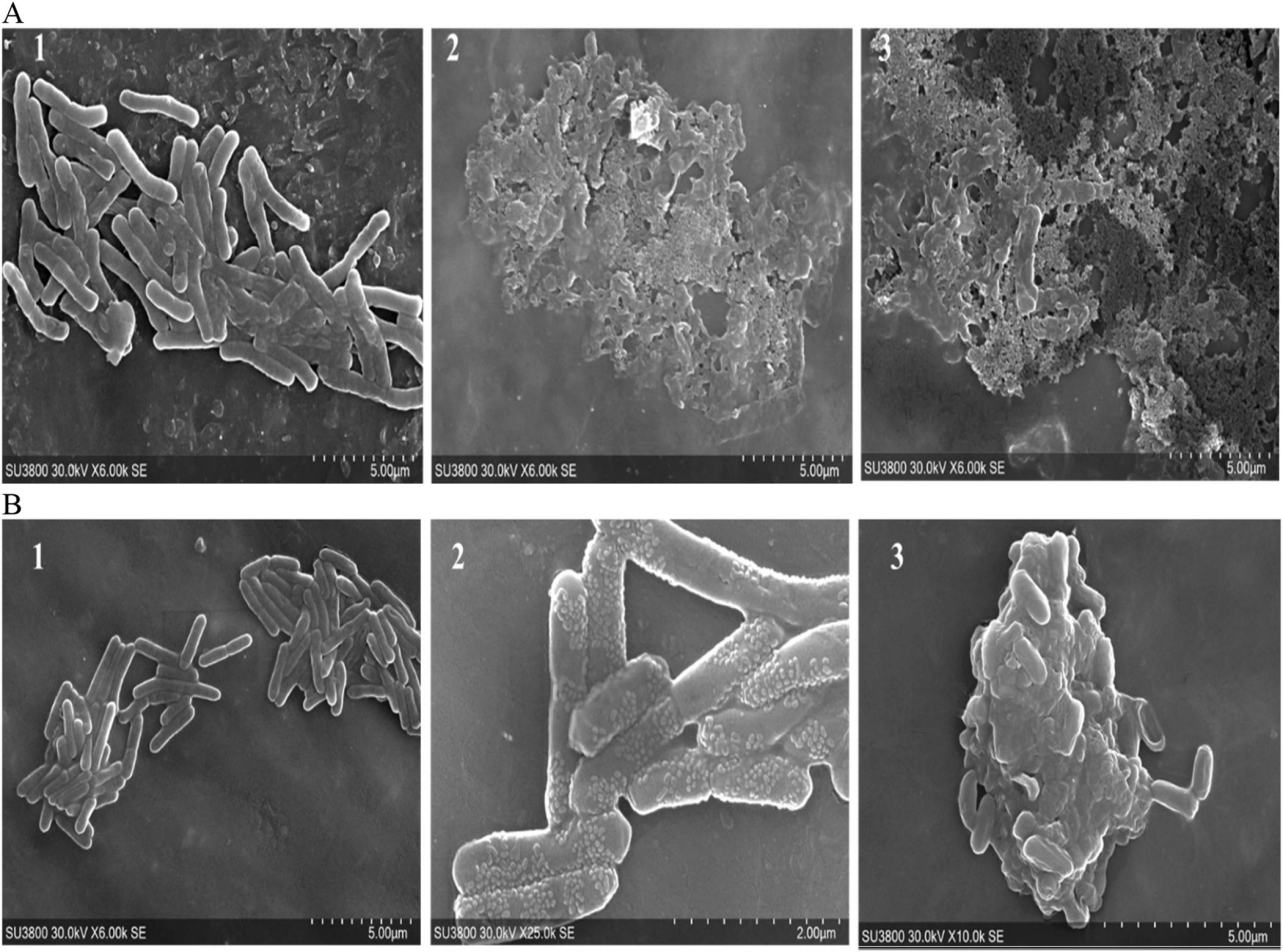
Scanning electron micrographs of (A) *B. subtilis* MTCC 121 ^T^ and (B) *B. pumilus* MTCC 1640 ^T^ cells: (1) control (untreated cells with buffer instead of lysin), (2) bacterial cells treated with P19_358 lysin, and (3) Art2. Magnification: up to 25,000×. The scale bar is represented in the bottom right corner.

SEM analysis was performed to evaluate the morphological changes in *B. subtilis* MTCC 121 ^T^ and *B. pumilus* MTCC 1640 ^T^ cells following enzymatic treatment. *B. subtilis* MTCC 121 ^T^ was selected as the most sensitive species to lysin, while *B. pumilus* MTCC 1640 ^T^ was chosen as the least sensitive among the tested *Bacillus* species. Untreated control cells of both *B. subtilis* MTCC 121 ^T^ and *B. pumilus* MTCC 1640 ^T^ exhibited intact morphology with smooth, intact, and well‐defined cell surfaces, indicating healthy and undamaged cells (Figure 6A1 and 6B1). Upon treatment with P19_358 lysin, *B. subtilis* MTCC 121 ^T^ cells showed complete lysis, with extensive cellular debris visible Figure 6A2), whereas *B. pumilus* MTCC 1640 ^T^ cells displayed only minor alterations in cell surface morphology, consistent with its limited susceptibility to the native P19_358 lysin (Figure 6B2). In contrast, treatment with Art2 resulted in complete lysis of *B. subtilis* MTCC 121 ^T^ (Figure 6A3), as indicated by the absence of intact cells and the presence of disrupted cellular debris. Art2 treatment resulted in extensive cell damage, aggregation, and significant morphological disruption of *B. pumilus* MTCC 1640 ^T^ cells (Figure 6B3).

## 4 Discussion

The increasing prevalence of antimicrobial resistance has intensified the search for alternative antibacterial agents, particularly protein‐based antimicrobials such as phage endolysins and engineered artilysins (Briers et al. 2014). In this study, we report the rational design, expression, and functional characterization of novel artilysins derived from the P19 endolysin. This P19_358 lysin is encoded by a lytic phage P19, previously isolated from the Ganga River, using *A. dhakensis* CECT 5744 ^T^ as the host. P19_358 lysin is a T4‐type lysozyme (IPR001165) belonging to glycoside hydrolase family 24, encoded by ORF_358 of phage P19. Its conserved domain belongs to the baseplate hub subunit and tail lysozyme family, and shares evolutionary relationships with endolysins from *Aeromonas* phages. This enzyme exhibits muralytic activity exclusively against *B. subtilis* MTCC 121 ^T^ among the tested Gram‐positive bacteria without the use of EDTA, and shows activity against Gram‐negative bacteria (*A. dhakensis* (CECT 5744^T^, LMG 24688, and A2), *Escherichia coli* MTCC 1625, *Shigella flexneri* MTCC 1457, *Salmonella* Typhimurium MTCC 98, and *Pseudomonas aeruginosa* NCIM 2036) only upon pre‐treatment with 0.1 M EDTA (Ansari and Nagar 2026). This limitation is consistent with the inability of native endolysins to penetrate the outer membrane of Gram‐negative bacteria (Briers et al. 2014). Endolysins targeting Gram‐positive bacteria have been extensively studied and several have advanced into clinical development (Borysowski et al. 2006) or are being explored in food industrial applications (Abdelkader et al. 2022; Li et al. 2022), whereas progress against Gram‐negative pathogens has been comparatively limited. To expand endolysin applicability, protein engineering has been explored (Briers et al. 2014).

In this study, we employed a protein engineering strategy to enhance the antibacterial spectrum and efficacy of P19_358 lysin by fusing it with two distinct polycationic, amphipathic peptides, Cecropin A and a polycationic nonapeptide (PCNP), with and without flexible glycine‐serine linkers. Cecropin A is a 37‐amino acid cationic peptide originally isolated from the moth *Hyalophora cecropia*. It binds to negatively charged bacterial membranes through its positively charged N‐terminal region. A flexible hinge region allows the peptide to insert into the membrane, leading to pore formation or membrane disruption, which ultimately kills the bacteria (Abdelkader et al. 2022). Shortened derivatives of Cecropin A (KWKLFKKI), an 8‐amino acid peptide, are often used in engineering. These short peptides themselves lack antimicrobial activity but enhance the ability of endolysins to bind the bacterial membrane by dissipating the electrochemical gradient across the cytoplasmic membrane (Wojciechowska 2025; Yang et al. 2020). PCNP (KRKKRKKRK) is another short, positively charged peptide that binds to the bacterial membrane to facilitate the access of lysins (Briers et al. 2014). The rationale behind C‐terminal fusion was to preserve the native catalytic domain of the endolysin while enhancing membrane penetration and surface binding.

Three‐dimensional structural comparison of the native P19_358 lysin and engineered artilysins revealed that C‐terminal peptide fusion did not induce any detectable interference in the core catalytic domain or active site architecture. Structural alignment demonstrated strong conservation of the endolysin fold, indicating that artilysin engineering preserved the intrinsic enzymatic framework required for peptidoglycan hydrolysis (Supplementary Figure S1). This observation is also supported by physicochemical studies, including CD and fluorescence spectroscopy, indicating no change in the structure.

Functional characterization revealed that artilysin modification markedly improved both the antibacterial efficacy and host spectrum of the native P19_358 lysin (Figure 2 and Supplementary Table S1). While the native endolysin displayed limited activity, primarily restricted to *B. subtilis* MTCC 121 ^T^, the engineered artilysins exhibited pronounced lytic activity against multiple *Bacillus* species (*B. pumilus* MTCC 1640 ^T^ and *B. licheniformis* MTCC 429 ^T^). Antibacterial activity assessed using both turbidity reduction and CFU enumeration assays consistently demonstrated that Art1 and Art2 were the most potent artilysins, indicating that peptide selection and fusion strategy critically influence artilysin performance. Notably, both Art1 and Art2 incorporate Cecropin A‐derived peptides, highlighting the effectiveness of this cationic peptide in artilysin design. The enhanced activity observed for Art1 and Art2, both of which include flexible glycine‐serine linkers, suggests that linker flexibility facilitates optimal spatial orientation between the catalytic domain and the membrane‐active peptide, thereby improving bacteriolytic efficiency. These findings are consistent with previous reports demonstrating the benefits of Cecropin A fusion in engineered lysins. Islam et al. (2022) reported a 2–8‐fold increase in antibacterial activity of a Cecropin A‐fused *Acinetobacter baumannii* endolysin compared to its native counterpart (Islam et al. 2022). Similarly, Yang et al. (2015) also demonstrated enhanced lytic activity of a Cecropin A‐modified endolysin (PlyA) (Yang et al. 2015). In addition, PCNP‐modified artilysins in the present study also exhibited enhanced antibacterial activity, with slower kinetics, indicating that peptide composition influences both potency and temporal dynamics of bacterial killing (Figure 2 and Supplementary Figure S4A–C). Variability among bacterial species was observed in susceptibility to artilysin treatment. *B. subtilis* MTCC 121 ^T^ showed the highest level of killing (up to 5.4 log_10_ reduction), likely due to relatively fewer structural modifications in its cell wall, making the peptidoglycan more accessible. In contrast, *B. pumilus* MTCC 1640 ^T^ and *B. licheniformis* MTCC 429 ^T^ exhibited lower killing efficiency (up to 4.9 log_10_ reduction), which may be due to differences in cell wall composition and architecture. *B. pumilus* is reported to have higher levels of N‐deacetylation and O‐acetylation of peptidoglycan sugars, modifications that can reduce the susceptibility to lytic enzymes (Kojima et al. 1985). Similarly, *B. licheniformis* can produce secondary cell wall polymers that can hinder the access of artilysin to the peptidoglycan layer (Hughes 1970). In addition, no significant difference in antibacterial activity was observed between P19_358 lysin and Art2 against EDTA‐pre‐treated Gram‐negative bacterial cells.

Biofilm‐associated infections pose a major challenge due to the intrinsic tolerance of biofilm‐embedded bacteria to antimicrobial agents (Zhao et al. 2023). In this study, Art2 demonstrated pronounced antibiofilm activity, significantly reducing biofilm formation across all tested *Bacillus* species (Figure 3). In contrast, the native P19_358 lysin exhibited limited antibiofilm efficacy, where biofilm formation was comparable to untreated controls. The enhanced antibiofilm performance of Art2 is likely attributable to its superior bactericidal activity, resulting in a reduced number of viable cells available for biofilm initiation and maturation. This correlation between bacterial cell killing and biofilm inhibition has also been reported for other artilysins like AL‐3AA modified from LysPA26 (Wang et al. 2021), underscoring the importance of early bacterial elimination in preventing biofilm establishment. In contrast, the lysin fails to effectively disrupt mature biofilms, the limited activity may be attributed to the protective extracellular polymeric matrix, which restricts enzyme penetration. The lytic activity of the engineered artilysin was further confirmed through SEM, which revealed extensive morphological damage in treated bacterial cells (Figure 6). The presence of completely lysed cells accompanied by prominent cellular debris indicates efficient disruption of the bacterial cell wall. Such structural disintegration is characteristic of the anti‐bacterial enzymatic activity, also reported by Briers et al. (2014), in which artilysin caused severe membrane destabilization and cell wall degradation, resulting in visible cellular collapse and debris formation under electron microscopy.

Among all artilysins, Art2 consistently exhibited the highest antibacterial activity and biofilm reductions among tested strains, prompting its selection for stability analyses in comparison with the native P19_358 lysin. Thermal stability assays demonstrated that Art2 retained maximal activity across a temperature range of 4°C–45°C and maintained approximately 50% residual activity even at 55°C. Across almost all tested temperatures, Art2 showed superior stability relative to the native endolysin, suggesting that peptide fusion may impart additional structural resilience. Similar temperature stabilization effects following peptide engineering have been reported for other artilysins. Islam et al. (2022) reported enhanced thermal stability in cecropin A‐modified eAbEndolysin and AbEndolysin across a temperature range of 4°C–42°C (Islam et al. 2022), comparable to the stability observed in Art2 in the present study. Both P19_358 lysin and Art2 exhibited optimal activity under mildly acidic conditions, with maximal stability observed between pH 5 and 7. This observation was also supported by DSF analysis, showing the highest Tm at pH 6 for all the artilysins and P19_358 lysin as well. These findings align well with a previous report demonstrating P19_358 lysin stability within a pH range of 5.5–7 using dynamic light scattering analysis (Ansari and Nagar 2026). Interestingly, although Art2 exhibited a lower overall thermal unfolding temperature compared to the parental lysin, it retained substantial antibacterial activity at 45°C, indicating that thermal unfolding and functional activity may not always directly correlate. The retained activity of Art2 at elevated temperature may result from enhanced functional stability, improved substrate interaction, or partial preservation of active‐site conformation despite partial structural destabilization. Notably, Art2 retained higher relative activity than the native P19_358 lysin almost across all tested pH spectrum (pH 2–12). Similar observations have been reported for other artilysins, including Art‐240, modified from λSa2lys with a PCNP chain (Rodríguez‐Rubio et al. 2016), and cecropin A‐modified eAbEndolysin, which retained activity across a pH range of 5.5–7.5 (Islam et al. 2022), collectively indicating that artilysin engineering broadens the functional pH range of the native enzyme. Art2 retained more than 90% of its relative activity in the presence of NaCl concentrations up to 0.3 M, demonstrating significantly higher salt tolerance compared to P19_358 lysin. In contrast, other *Bacillus cereus* infecting phage endolysins, such as PlyB13S endolysin, have been reported to exhibit a substantial decline in activity, with more than 50% loss of activity observed at 0.1 M NaCl (Yu and Kong 2025a).

Collectively, these findings demonstrate that rational artilysin engineering through C‐terminal peptide fusion significantly enhances antibacterial spectrum, stability, and antibiofilm efficacy while preserving the native endolysin catalytic structure. Art2, in particular, emerges as a promising candidate for further development as a robust protein‐based antimicrobial agent. The enhanced performance of Art2 underscores the importance of construct architecture, including linker selection and fusion orientation, in determining functional outcomes. Importantly, the observed activity against multiple *Bacillus* species suggests that these engineered artilysins may have broad applicability in food safety, industrial sanitation, and potentially clinical settings. However, this study has certain limitations, as the antibacterial activity was evaluated only against a limited number of *Bacillus* species. Therefore, further studies involving clinically relevant pathogenic *Bacillus* species, along with additional optimization of peptide composition, linker architecture, and domain organization along with mechanistic studies on host specificity will be necessary to broaden the antibacterial spectrum and better understand the host specificity of these engineered artilysins.

## 5 Conclusion

This study demonstrates the successful cloning, expression, and rational engineering of an *A. dhakensis* phage‐derived P19_358 lysin into potent artilysins. While the native enzyme exhibited narrow lytic activity against *B. subtilis* MTCC 121 ^T^, C‐terminal fusion with a cationic peptide significantly expanded its antibacterial spectrum to include *B. pumilus* MTCC 1640 ^T^ and *B. licheniformis* MTCC 429 ^T^, achieving > 3‐log CFU reductions at low concentrations (5.3 µM). The engineered artilysin also displayed robust thermal stability (up to 50°C for 30 min) and superior antibiofilm efficacy, disrupting *Bacillus* biofilms by 70–85% in 24 h assays. These findings validate peptide fusion as an effective strategy to overcome the host‐range limitations of native endolysins and generate a lysin with an enhanced lytic spectrum. The artilysin's stability, potency, and biofilm‐disrupting capacity position it as a promising candidate for non‐antibiotic biocontrol in food processing (dairy and meat decontamination), aquaculture pathogen management, and therapeutic applications against multidrug‐resistant *Bacillus* infections. Future optimization should focus on in vivo efficacy, scalability, and regulatory pathway assessment to accelerate clinical and industrial translation.

## Author Contributions

**Farhat Ansari:** conceptualization, methodology, investigation, formal analysis, writing – original draft. **Tarushi:** methodology, investigation, and formal analysis. **Vandan Nagar:** conceptualization, methodology, writing – review and editing, formal analysis, supervision.

## Ethics Statement

Not applicable. This study involved *in‐vitro* and *in‐silico* analysis of a phage‐encoded endolysin and its modification into artilysins by genetic engineering, and did not involve human participants, animal experimentation, or any biological material requiring ethical approval.

## Conflicts of Interest

The authors declare no conflicts of interest.

## Supporting information

Supporting File

## Data Availability

The data that support the findings of this study are available from the corresponding author upon reasonable request.
