# Supplementary material for: Polycationic Peptide Engineering of Phage Endolysins Expands Host Range and Enhances Antibacterial and Antibiofilm Activities Against Bacillus Species
Source: Biotechnol Bioeng. 2026 Jul 6;123(10):2788–801. doi: 10.1002/bit.70293 (PMC13576789; doi:10.1002/bit.70293)
Supplement: Supplementary file 1 [file BIT-123-2788-s001.docx]

Table S1: Details of bacterial cultures and host range analysis of phage P19, P19_358 lysin, and artilysins (without EDTA pre-treatment) by spot test.

|  |  | **Bacterial strain** | **Source** | **Phage P19** | **P19_358 lysin** | **All artilysins** |
| --- | --- | --- | --- | --- | --- | --- |
| Gram Negative | *Aeromonas dhakensis* | CECT^a^ 5744^T^ | Stool | + | - | - |
|  | *A. dhakensis* | LMG 24688^b^ | Fish | + | - | - |
|  | *A. dhakensis* | A2^c^ | River | + | - | - |
|  | *Salmonella* Typhimurium | MTCC^d^ 98 | Clinical | - | - | - |
|  | *Pseudomonas aeruginosa* | NCIM^e^ 2036 | Pond Water | - | - | - |
|  | *Escherichia coli* | MTCC 1625 | Food sample | - | - | - |
|  | *Shigella flexneri* | MTCC 1457 | River water | - | - | - |
| Gram Positive | *Bacillus subtilis* | MTCC 121^T^ | Soil | - | **+** | + |
|  | *Bacillus licheniformis* | MTCC 429^T^ | Soil | - | - | + |
|  | *Bacillus pumilus* | MTCC 1640^T^ | Soil | - | - | + |
|  | *Bacillus cereus* | NCTC^f^ 10320 | Reference Strain | - | - | - |
|  | *Staphylococcus aureus* | MTCC 1430^T^ | Clinical | - | - | - |
|  | *Micrococcus luteus* | MTCC 106^T^ | Soil | - | - | - |
|  | *Lactococcus lactis* | MTCC 1484^T^ | Dairy | - | - | - |
|  | *Lactobacillus rhamnosus* | MTCC 1408^T^ | Dairy | - | - | - |
|  | *Lactobacillus plantarum* | MTCC 2621 | Dairy | - | - | - |
|  | *Lactobacillus fermentum* | MTCC 9748 | Dairy | - | - | - |
|  | *Rhodococcus rhodochrous* | MTCC 265^T^ | Soil | - | - | - |
|  | *Listeria monocytogenes* | MTCC 657 | Poultry | - | - | - |

^a^CECT - Spanish Type Culture Collection

^b^LMG- Laboratory of Microbiology, Ghent University

^c^LS- Lab stock (Nagar et al. 2025)

^d^MTCC - Microbial Type Culture Collection, India

^e^NCIM - National Collection of Industrial Microorganisms

^f^NCTC - National Collection of Type Cultures, UK

Table S2: Details of primer sequences. Changes in artilysin primers as compared to P19_358 lysin are highlighted in bold.

| **Artilysin** | **Primer sequences** |
| --- | --- |
| P19_358 lysin | (Forward) 5′-GGTTCGGATCCATGTTAGAAAAAATGCTTAAATTCG-3′  (Reverse) 5′-GGAATTCCTCGAGTTATGCGAATGGATAACCTTTG-3′ |
| Art1 | (Forward) 5′-GGTTCGGATCCATGTTAGAAAAAATGCTTAAATTCG**ACGAGGG**-3′  (Reverse) 5′-GGAATTCCTCGAGTTA**GATTTTCTTGAACAGTTTCCATTTCGCGCTGCCTGCGAATGGATAACCTTTGTAAG**-3′ |
| Art2 | (Forward) 5′-GGTTCGGATCCATGTTAGAAAAAATGCTTAAATTCG**ACGAGGG**-3′  (Reverse) 5′-GGAATTCCTCGAGTTA**GATTTTCTTGAACAGTTTCCATTTGCTGCCGCTGCCTGCGAATGGATAACCTTTGTAAG**-3′ |
| Art3 | (Forward) 5′-GGTTCGGATCCATGTTAGAAAAAATGCTTAAATTCG**ACGAGGG**-3′  (Reverse) 5′-GGAATTCCTCGAGTTA**GATTTTCTTGAACAGTTTCCATTTTGCGAATGGATAACCTTTGTAAG**-3′ |
| Art4 | (Forward) 5′-GGTTCGGATCCATGTTAGAAAAAATGCTTAAATTCG**ACGAGGG**-3′  (Reverse) 5′- GGAATTCCTCGAGTTA**TTTGCGTTTTTTGCGTTTTTTGCGTTTTGCGAATGGATAACCTTTGTAAG**-3′ |
| Art5 | (Forward) 5′-GGTTCGGATCCATGTTAGAAAAAATGCTTAAATTCG**ACGAGGG**-3′  (Reverse) 5′-GGAATTCCTCGAGTTA**TTTGCGTTTTTTGCGTTTTTTGCGTTTGCTGCCGCTGCCTGCGAATGGATAACCTTTGTAAG**-3′ |
| Art6 | (Forward) 5′-GGTTCGGATCCATGTTAGAAAAAATGCTTAAATTCG**ACGAGGG**-3′  (Reverse) 5′-GGAATTCCTCGAGTTA**TTTGCGTTTTTTGCGTTTTTTGCGTTTGCTGCCTGCGAATGGATAACCTTTGTAAG**-3′ |

Table S3: Three-dimensional structure alignment and superimposition of P19_358 lysin and artilysins. Low Root Mean Square Deviation (RMSD) values signify higher structure similarity. Template Modelling Score (TM-Score) and Maximum Similarity Score (MaxSub Score) range from 0 (least identical) to 1 (most identical).

|  | **RMSD (Core)** | **TM-score** | **MaxSub score** |
| --- | --- | --- | --- |
| **Art1** | 0.34 Å | 0.9344 | 0.9311 |
| **Art2** | 0.66 Å | 0.9166 | 0.9013 |
| **Art3** | 0.53 Å | 0.9430 | 0.9327 |
| **Art4** | 0.59 Å | 0.8213 | 0.7512 |
| **Art5** | 0.21 Å | 0.9258 | 0.9242 |
| **Art6** | 0.64 Å | 0.9225 | 0.9079 |

Fig. S1: Three-dimensional structure superimposition of native (P19_358 lysin) (red) and artilysins (grey): (A) Art1, (B) Art2, (C) Art3, (D) Art4, (E) Art5, and (F) Art6.

Fig. S2: Representative 0.8 % agarose gel of positive artilysin clones double digested by restriction enzyme *Bam*HI and *Xho*I. Lane 1: Art1; Lane 2: Art2; and Lane 3: Art3. Restriction digestion shows two fragments; larger one is linearized plasmid and smaller one represents the insert of size ~ 525 bp.

Fig. S3: 15% SDS-PAGE of IPTG-induced proteins. Lane 1: P19_358 lysin; Lane 2: Art1; M: protein ladder (kDa); Lane 3: Art2; Lane 4: Art3; Lane 5: Art4; Lane 6: Art5; and Lane 7: Art6.

Fig. S4: Spot test analysis for determining the host range of P19_358 lysin and artilysins.

Fig. S5: Relative lytic activity (%) of P19_358 lysin and artilysins by the turbidity reduction method at 20 min and 120 min compared to the control (buffer instead of protein). (A) *B. subtilis*, (B) *B. pumilus*, and (C) *B. licheniformis*. Statistical significance was determined by two-way ANOVA at p < 0.05. Different letters above the error bars represent a significant difference in lytic activity between the two time points (20 min and 120 min) with the same protein at p < 0.05, whereas the same letter represents no significant difference under the same condition.

(A)

(B)

(C)

Fig. S6: Relative lytic activity (%) of P19_358 lysin and Art2 by the turbidity reduction method at 120 min (buffer instead of protein). Statistical significance was determined by two-way ANOVA at p < 0.05. Same letters above the error bars represent a no significant difference in lytic activity under the same condition.

Fig. S7: Percentage of mature biofilm formation by *B. subtilis* MTCC 121^T^ treated with P19_358 lysin and artilysins determined by the crystal violet method. Statistical significance was determined by two-way ANOVA at p < 0.05.

References:

1. Nagar, V., Ansari, F., Vaiyapuri, M., & Joseph, T. C. (2025). Virulent and multidrug-resistant *Aeromonas* in aquatic environments of Kerala, India: potential risks to fish and humans. Brazilian Journal of Microbiology, 56(1), 303–311. https://doi.org/10.1007/s42770-024-01601-w.
